# The Effect of Serine Protease Inhibitors on Visceral Pain in Different Rodent Models With an Intestinal Insult

**DOI:** 10.3389/fphar.2022.765744

**Published:** 2022-06-02

**Authors:** Hannah Ceuleers, Nikita Hanning, Michelle De bruyn, Joris G De Man, Heiko U De Schepper, Qian Li, Liansheng Liu, Steven Abrams, Annemieke Smet, Jurgen Joossens, Koen Augustyns, Ingrid De Meester, Pankaj J Pasricha, Benedicte Y De Winter

**Affiliations:** ^1^ Laboratory of Experimental Medicine and Pediatrics, University of Antwerp, Antwerp, Belgium; ^2^ Center for Neurogastroenterology, Department of Medicine, Johns Hopkins University School of Medicine, Baltimore, MD, United States; ^3^ Infla-Med, Centre of Excellence, University of Antwerp, Antwerp, Belgium; ^4^ Laboratory of Medical Biochemistry, University of Antwerp, Antwerp, Belgium; ^5^ Department of Gastroenterology and Hepatology, Antwerp University Hospital, Antwerp, Belgium; ^6^ Global Health Institute, University of Antwerp, Antwerp, Belgium; ^7^ Data Science Institute, UHasselt, Hasselt, Belgium; ^8^ Laboratory of Medicinal Chemistry, University of Antwerp, Antwerp, Belgium

**Keywords:** serine protease inhibitor, visceral hypersensitivity, rodent models, inflammatory bowel diseases, irritable bowel syndrome, proteolytic activity, protease profiling

## Abstract

**Background:** Serine proteases are believed to play a key role in the origin of abdominal pain in IBD and IBS. We previously demonstrated a reduction of visceral pain in a post-inflammatory IBS rat model after a single intraperitoneal or intracolonic administration of a serine protease inhibitor. The aim of this study was to investigate the efficacy of serine protease inhibition on visceral pain in two different animal models involving a colonic insult based either on acute inflammation or on neonatal irritation. Moreover, protease profiling was explored in the acute colitis model.

**Methods:** An acute 2,4,6-trinitrobenzenesulphonic acid (TNBS) colitis rat model and a chronic neonatal acetic acid mouse model were used in this study. Visceral sensitivity was quantified by visceromotor responses (VMRs) to colorectal distension, 30 min after intraperitoneal administration of the serine protease inhibitors nafamostat, UAMC-00050 or their vehicles. Colonic samples from acute colitis rats were used to quantify the mRNA expression of a panel of serine proteases and mast cell tryptase by immunohistochemistry. Finally, proteolytic activities in colonic and fecal samples were characterized using fluorogenic substrates.

**Key Results:** We showed a significant and pressure-dependent increase in visceral hypersensitivity in acute colitis and neonatal acetic acid models. UAMC-00050 and nafamostat significantly reduced VMRs in both animal models. In acute colitis rats, the administration of a serine protease inhibitor did not affect the inflammatory parameters. Protease profiling of these acute colitis animals revealed an increased tryptase immunoreactivity and a downregulation of matriptase at the mRNA level after inflammation. The administration of UAMC-00050 resulted in a decreased elastase-like activity in the colon associated with a significantly increased elastase-like activity in fecal samples of acute colitis animals.

**Conclusion:** In conclusion, our results suggest that serine proteases play an important role in visceral hypersensitivity in an acute TNBS colitis model in rats and a neonatal acetic acid model in mice. Moreover, we hypothesize a potential mechanism of action of UAMC-00050 via the alteration of elastase-like proteolytic activity in acute inflammation. Taken together, we provided fundamental evidence for serine protease inhibitors as a promising new therapeutic strategy for abdominal pain in gastrointestinal diseases.

## 1 Introduction

Visceral hypersensitivity can be defined as an increased perception of stimuli arising from the intestines and is an important mechanism underlying abdominal pain. Abdominal pain is a common symptom in patients suffering from irritable bowel syndrome (IBS) and inflammatory bowel diseases (IBD) such as Crohn’s disease and ulcerative colitis (Vermeulen et al., 2014; Aziz and Simrén, 2021). It is largely substantiated in the literature that IBD patients, both during active disease as well as in remission, frequently report IBS-like symptoms (De Schepper et al., 2008; Halpin and Ford, 2012). Both disorders are regarded as an important healthcare problem because of their chronic character, the high prevalence, the considerable impact on the patients’ quality of life and the association with a high socio-economic burden (Lovell and Ford, 2012; Ananthakrishnan, 2015; Oka et al., 2020). Besides, the currently available therapeutic strategies mostly aim at reducing the inflammation in IBD patients or the predominant motility disturbance in IBS patients. This is in sharp contrast with the limited amount of therapies that are available to directly target abdominal pain (Camilleri, 2021). Furthermore, pain treatment in these patients is challenging due to the known side effects of classical analgesics and their potential to even exacerbate symptoms (Norton et al., 2017). Hence, additional studies towards the search for new treatment targets in the domain of visceral pain are of great interest but unfortunately hampered due to the incomplete comprehension of the pathophysiological mechanisms involved. Furthermore, a more personally tailored treatment would be beneficial for IBD and IBS patients (Simrén and Tack, 2018; Digby-Bell et al., 2020).

In this respect there is strong evidence for the implication of the serine protease pathway in the pathophysiology of visceral hypersensitivity (Cenac et al., 2007; Ceuleers et al., 2016; Rolland-Fourcade et al., 2017; Ceuleers et al., 2018; Desormeaux et al., 2018; Jimenez-Vargas et al., 2018; Hanning et al., 2021). Proteases are present at particularly high levels in the gastrointestinal tract and important sources include the pancreas, intestinal microbiota, the epithelium, macrophages, inflammatory cells, e.g., neutrophils and mast cells (Vergnolle, 2016). Indeed, mast cells have been shown to fulfill an important task in the development of abdominal pain in IBS patients (De Winter et al., 2012), besides an association was found between activated mast cells in close proximity to colonic nerves and the frequency and the severity of abdominal pain (Barbara et al., 2004) and a mast cell-sensory neuron adhesion was recently put forward (Magadmi et al., 2019). The stabilization of mast cells by using histamine H1 and H4 receptors have showed promising results in IBS in both preclinical and clinical settings (Deiteren et al., 2014; Wouters et al., 2016). Moreover, interference with the serine protease pathway via protease-activated receptors (PAR) has been put forward as a possibly interesting option for the treatment of visceral pain, since multiple *in vitro* and *in vivo* studies demonstrated beneficial effects using different PAR-agonists and antagonists (Ceuleers et al., 2016). However, none of these compounds eventually made it to the clinic so far. In more recent years, an intervention more upstream of this PAR-activating cascade, namely a direct inhibition of proteases, has been suggested (Vergnolle, 2016; Augustyns et al., 2017). Up until now, only a few preclinical animal studies demonstrated the *in vitro* (Cenac et al., 2007; Wang et al., 2015) or *in vivo* (Zhao et al., 2011) effect of serine protease inhibitors on visceral pain. Additionally we previously demonstrated a reduction of visceral pain in a post-inflammatory IBS rat model after a single intraperitoneal (i.p.) (Ceuleers et al., 2018) or intracolonic (Hanning et al., 2021) administration of different serine protease inhibitors, including a new class of serine protease inhibitors developed at the University of Antwerp (Joossens et al., 2007). After demonstrating these beneficial effects of serine protease inhibition on visceral pain in a post-inflammatory rat model for IBS we aimed to substantiate these findings in other animal models.

The aim of this study was to investigate the efficacy of a pharmacological intervention using the serine protease inhibitors nafamostat mesylate and UAMC-00050 in two different animal models of visceral pain involving a colonic insult based on inflammation on the one hand and neonatal irritation on the other hand. We therefore used an acute TNBS colitis model in the rat resembling pain in an acute phase of inflammation (IBD) (Vermeulen et al., 2013; Deiteren et al., 2015) and a neonatal acetic acid-induced model in mice for IBS (Winston et al., 2007; Li et al., 2014), set up at the University of Antwerp and the Johns Hopkins University, respectively. In parallel, we assessed the serine protease profile in the colon of acute colitis rats and investigated the effect of treatment with UAMC-00050 on the serine protease activities in the colon and feces of acute colitis rats.

## 2 Materials and Methods

### 2.1 Animals

Male Sprague-Dawley rats (200–225 g; Charles River, Calco, Italy) were used based on previous studies (Deiteren et al., 2014; Deiteren et al., 2015; Ceuleers et al., 2018) and housed at constant room temperature (22 ± 2°C) and humidity (60%) with two rats per cage. Rats had unlimited access to water and food and were kept on a 12h:12h day-night cycle. All experiments were approved by the Ethical Committee for Animal Experiments of the University of Antwerp (EC nr. 2014-41).

C57BL/6 mice (8–12 weeks old, male and female) were originally purchased from Charles River Laboratories, United States. Breeding was maintained at the Johns Hopkins University, Baltimore, MD, United States. Mice were housed with five animals per cage at constant room temperature (23°C) and humidity (45%). They had unlimited access to water and food and were kept on a 12h:12h day-night cycle. All experiments were approved by the Institutional Animal Care and Use Committee (IACUC) of the Johns Hopkins University (M016M107).

### 2.2 Acute TNBS Colitis Rat Model and Experimental Design

2,4,6-trinitrobenzenesulphonic acid (TNBS) colitis was induced as previously described at day 0 using a TNBS-enema (4 mg TNBS, 50% ethanol) (Deiteren et al., 2014; Deiteren et al., 2015; Ceuleers et al., 2018; Hanning et al., 2021). After an overnight fast, 0.25 ml of the TNBS-enema was administered intrarectally using a flexible catheter (18G, length 4.5 cm), under anesthesia with a mixture of ketamine (35 mg/kg i. p.) and xylazine (5 mg/kg i. p.). Control animals received an enema containing 0.25 ml 0.9% NaCl. The animals were kept in a tail-up position during 1 min and were then allowed to recover in a Trendelenburg position with free access to water and food. On day 3, the presence of acute colitis was confirmed by colonoscopy in every individual animal (Ceuleers et al., 2018).

**FIGURE 1:**
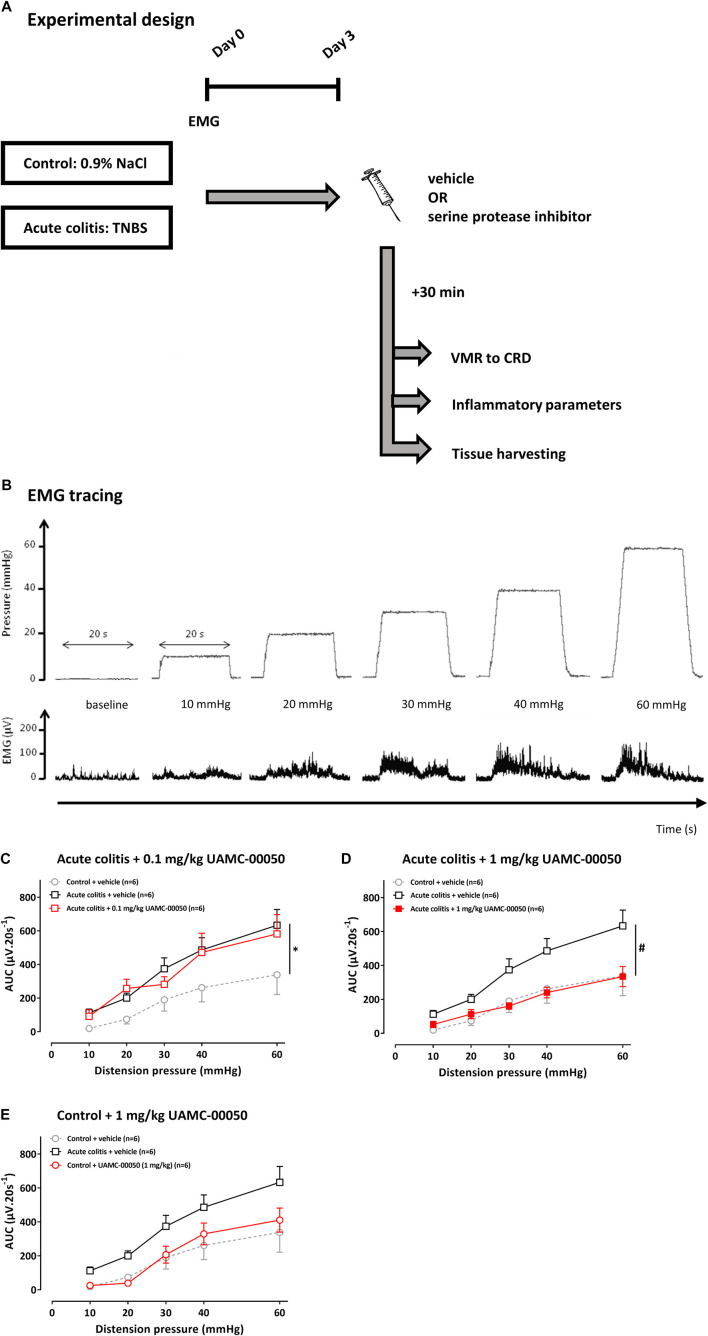
The effect of UAMC-00050 (0.1–1 mg/kg) and its vehicle (5% DMSO) on VMRs in acute colitis and control rats. **(A)** Overview of the experimental design in acute TNBS-colitis rats. On day 0, rats had an intrarectal administration with TNBS (colitis) or saline (control) and the implantation of EMG electrodes. Further experiments were conducted at day 3. A compound (nafamostat or UAMC-00050) or vehicle (5% DMSO) was injected i. p. 30 min prior to the VMR experiment. Afterwards, the inflammatory status was determined and tissues were harvested for further experiments. CRD: colorectal distension; EMG; electromyographic; TNBS; 2,4,6-trinitrobenzenesulfonic acid; VMR; visceromotor response. **(B)** Example of a processed EMG tracing (DC remove (time constant 0.1 s), full-wave rectification and smoothening (time constant 0.01 s); µV), presented for each distension pressure (mmHg). **(C–E)** The effect of UAMC-00050 (0.1–1 mg/kg) and its vehicle (5% DMSO) on VMRs in acute colitis (squares) and control (bullets) rats. The statistical analysis was performed on the complete dataset, but separate graphs were made for each dose for the purpose of clarification. Data are presented as mean ± SEM. Generalized Estimating Equations (GEE) + Bonferroni-Holm correction to correct for multiple testing and control for the family-wise error rate across model-specific analyses. n = 6/group; *p <0.05; significantly different from control + vehicle. #*p* <0.05; significantly different from acute colitis + vehicle.

In the acute TNBS rat model, experiments were performed 3 days after induction of colitis (Vermeulen et al., 2011). An overview of the experimental course is shown in Figure 1A. Rats were administered UAMC-00050 (0.1 or 1 mg/kg), nafamostat (0.1, one or 10 mg/kg) or vehicle (5% DMSO) i. p. 30 min before the start of the VMR experiment. These doses were chosen according to the beneficial effects of these compounds in rats in the post-colitis model (Ceuleers et al., 2018). Afterwards, the animals were anesthetized to perform a colonoscopy and after sacrifice the following post-mortem inflammatory parameters (macroscopy, microscopy and myeloperoxidase activity (MPO)) were scored. Colonic samples were taken from another group of control and acute colitis animals for further qPCR and immunohistochemistry experiments (n = 8/group). Colonic and fecal samples were obtained from vehicle- and UAMC-00050-treated acute colitis rats to assess proteolytic activities using fluorogenic substrates (n = 6–8/group).

### 2.3 Acetic Acid-Induced Mouse Model and Experimental Design

The neonatal acetic acid-induced IBS mouse model was previously described in literature as a model for visceral hypersensitivity albeit using different dose- and time regimens (Winston et al., 2007; Li et al., 2014; Zhang et al., 2020; Shaidullov et al., 2021). The experimental design we used is shown in Figure 3A. The infusion of 20 μL diluted acetic acid (0.5%) in neonatal mice results in a mild chemical irritation of the colon. In a similar model in rats it was previously shown that histological signs of inflammation on H&E staining of the colon of both pups and adult rats were absent and that there was no increase in MPO values and pro-inflammatory cytokines (IL-1α, IL-1β, IL-2, IL-6, TNF-α) at the colonic level (Winston et al., 2007). This early-life stress event eventually leads to the presence of visceral hypersensitivity in rodents in their adulthood (age 8–12 weeks) (Winston et al., 2007; Li et al., 2014).

Briefly, at postnatal day 10, C57BL/6 mice were intracolonically infused with 20 μL of a 0.5% acetic acid (AA) solution in 0.9% NaCl. Control animals received an infusion with 0.9% NaCl. Mice were weaned at the age of 21 days and used to assess visceral sensitivity at 8–12 weeks old.

Visceral sensitivity was assessed by means of the visceromotor responses (VMR) to colorectal distension at the age of 8–12 weeks old. Mice received a single i.p. injection with either vehicle (1% DMSO in sterile water), UAMC-00050 (2 mg/kg) or nafamostat mesylate (0.2 mg/kg) 30 min prior to the VMR experiment. All doses were based on the previously proven most effective i. p. doses in a TNBS-induced post-inflammatory rat model for IBS (Ceuleers et al., 2018) and have taken into account the necessary dose conversion due to differences in the metabolic rates between rats and mice. We used a table displaying approximate conversion factors for different species based on the equivalent surface area (Nair and Jacob, 2016). This table indicates a factor x2 when converting doses expressed as mg/kg from rats to mice. The animals were randomized and the investigators were blinded to the treatment.

### 2.4 Measurement of the Visceromotor Response

The visceromotor response (VMR) can be defined as a nociceptive reflex with a contraction of the abdominal muscles in response to a colorectal balloon distension (CRD) (Ness and Gebhart, 1988). The VMR is a validated, objective method, to quantify visceral sensitivity (Vermeulen et al., 2013; Deiteren et al., 2014; Deiteren et al., 2015; Greenwood-Van Meerveld et al., 2015; Ceuleers et al., 2018).

In the mouse, VMR to CRD was evaluated by EMG electrodes (Cooner Wire stranded stainless steel AS 631) that were implanted into the abdominal musculature 5 days prior to VMR assessment. At the day of the VMR registration and under isoflurane anesthesia (4% induction, 1.5% maintenance), a lubricated balloon (length 2 cm) was introduced into the colorectum. Afterwards, the mouse was put into a restrainer and allowed to recover from anesthesia for 30 min. Balloon distensions were induced according to the following pressure protocol: 15–30–50–70 mmHg, during 10 s, with at least 2 min intervals between different distensions. Each distension was performed twice, and the mean of these measurements was used for further analysis. The resulting EMG recordings were registered and analyzed using Spike2 version 5.16 (Cambridge Electronic Design, UK) for each pressure. The VMR signal was quantified as the modulus during distension (10 s), corrected by subtracting the modulus before distension (10 s, baseline).

In the rats, electromyographic (EMG) electrodes (Cooner wire G-32) were implanted into the external abdominal muscle of the rat 3 days prior to VMR assessment. On the day of the VMR experiment, a lubricated balloon (length 5 cm) was introduced into the colorectum of the conscious rat and connected to a barostat (Distender Series II Barostat, G&J Electronics, Canada) allowing phasic isobaric colonic distensions. The EMG electrodes were attached to a data-acquisition system and a phasic distension protocol (10–20–30–40–60 mmHg, 20 s, 4 min interval) was generated by the barostat. The EMG signal was registered, amplified (Neurolog, Digitimer Ltd., UK) and digitized (CED 1401, Cambridge Electronic Design, UK). The EMG signal (bandpass range 50 Hz–50 kHz) was further processed in Spike2 version 5.16 (Cambridge Electronic Design, UK) using the following parameters: DC remove (time constant 0.1 s), full-wave rectification and smoothening (time constant 0.01 s).

Then, the magnitude of the VMR signal was quantified at each distension pressure using the area under the curve (AUC) of the EMG signal during the distension (20 s) and normalized by subtracting the AUC of the EMG signal before the distension (20 s) (Figure 1B **)**.

### 2.5 Inflammatory Parameters in Acute Colitis Rats

#### 2.5.1 Colonoscopy

Under ketamine/xylazine anesthesia (35/5 mg/kg, i. p.), an *in vivo* colonoscopy was performed in the rats with a pediatric endoscope (Olympus GIF-N30, Olympus Europe GmbH), according to a previously published method (Vermeulen et al., 2011; Deiteren et al., 2014; Deiteren et al., 2015; Ceuleers et al., 2018). Briefly, the lubricated tip of the endoscope was introduced into the colon and advanced under endoscopic view until the hepatic flexure (±10 cm proximal to the anus) was reached. During withdrawal, the intestinal inflammation was evaluated using a standardized scoring system (total score 0-19) (Vermeulen et al., 2011; Deiteren et al., 2014; Deiteren et al., 2015; Ceuleers et al., 2018).

#### 2.5.2 Post-Mortem Inflammatory Markers

At the end of the experiments, the rats were sacrificed by exsanguination under pentobarbital anesthesia (45 mg/kg i. p). The colons were excised, rinsed with Krebs solution, opened along the mesenteric border and macroscopically evaluated using a validated scoring system (total score 0-10) (Vermeulen et al., 2011).

For microscopic evaluation, a colonic segment of approximately 1 cm^2^ was fixed in 4% formaldehyde for 24 h and embedded in paraffin for hematoxylin and eosin staining. The histological sections were given a microscopic score using a previously published scoring system (total score 0-10) (Vermeulen et al., 2011).

Finally, colonic myeloperoxidase (MPO) activity was determined as previously published (Vermeulen et al., 2011). MPO is a pro-inflammatory enzyme stored in the azurophilic granules of neutrophilic granulocytes and monocytes. Thus, the tissue MPO activity is a measure for the myeloid cell infiltration of the colonic wall (Pulli et al., 2013). The MPO activity is defined as the amount required to convert 1 µmol H_2_O_2_ to H_2_O within 1 min at 25°C and is expressed as unit/g tissue (Pulli et al., 2013).

### 2.6 Quantitative RT-PCR in Acute Colitis Rats

The mRNA expression levels of a panel of serine proteases was determined in colon samples of acute colitis and control rats by means of qPCR. Distal colonic segments were harvested from control and acute colitis rats, snap-frozen in liquid nitrogen and stored at −80°C. Total RNA was extracted from colon (Isolate II RNA Mini Kit, Bioline) and subsequently, RNA was converted to cDNA by reverse transcription (SensiFAST cDNA Synthesis Kit, Bioline). A Taqman gene expression assay (Thermofisher) was performed on an ABIPrism 7300 sequent detector system (Applied Biosystems, United States) in a 25 μl reaction volume containing 2 μl cDNA, 12.5 μl Taqman universal PCR Master Mix (ThermoFisher), 1.25 μl Taqman assay probe and 9.25 μl RNase-free H_2_O. The Taqman primers used were purchased from Thermofisher and are listed in Table 1. GAPDH and β-actin were used as reference genes. Gene expression assays were carried out according to the MIQE guidelines (Bustin et al., 2009). The PCR amplification parameters were 50°C for 2 min, 95°C for 10 min followed by 40 cycles of 95°C for 15 s and 60°C for 1 min (Bustin et al., 2009). The outcome values were analyzed using qBASE^PLUS^ software (Biogazelle N.V, Zwijnaarde, Belgium).

**TABLE 1:** Taqman primers used for qPCR analysis of colonic samples.

Protein	Gene id
Tryptase αβ1	Rn00570928_m1
Matriptase	Rn00586242_m1
Cathepsin G	Rn01489144_g1
Urokinase plasminogen activator	Rn00565261_m1
Kallikrein 2	Rn00820615_m1
Kallikrein 4	Rn01498534_g1
Kallikrein 8	Rn01476995_m1
GAPDH	Rn01775763_g1
β-actin	Rn00667869_m1

### 2.7 Immunohistochemistry in Acute Colitis Rats

Colonic samples of rats in the acute TNBS protocol were fixed in 4% formaldehyde, embedded in paraffin and cut in sections of 5 μm. Sections were pretreated with trypsin (37°C, 10 min) and citrate buffer pH6 (microwave, 10 min). Slides were incubated in a moist chamber with mouse mast cell tryptase monoclonal antibody (1:10.000, clone AA1, Abcam, Cambridge, United Kingdom) overnight. After a complete wash with Tris-saline buffer, slides were incubated with anti-mouse IgG antibody (1:200) and rat serum (1:20) for 30 min. Slides were then incubated with a Vectastain^®^ avidin-biotin complex (ABC) (Vector Laboratories, Burlingame, CA, United States) for 1 h. Subsequently, after a complete wash, the slides were developed in an aminoethylcarbazole (AEC) solution with hydrogen peroxide for 10 min and then counterstained with hematoxylin for 2 min and finally covered with glass. The colon sections were screened for mast cell tryptase positivity at ×100 magnification and for each layer (mucosa-submucosa-muscularis externa), the total number of tryptase positive cells was quantified using ImageJ 1.51 J8 (National Institutes of Health, United States) expressed per mm^2^.

### 2.8 Proteolytic Activities in Acute Colitis Rats

Trypsin-like, chymotrypsin-like, cathepsin G, elastase-like and kallikrein activities were assessed in colonic and fecal samples from acute colitis rats, according to a previously published method (Ceuleers et al., 2018; Hanning et al., 2021). ceulBriefly, distal colon and fecal samples were taken immediately after sacrifice of the animal, snap-frozen and stored at−80°C until further processing. The samples were crushed on dry ice, dissolved in lysis buffer and the enzymatic activity was measured by fluorescence spectroscopy on the supernatant using the following fluorogenic substrates: Boc-Gln-Ala-Arg-AMC and n-Tosyl-Gly-Pro-Arg-AMC for trypsin-like activity, Suc-Ala-Ala-Pro-Phe-AMC for chymotrypsin-like activity, Suc-Ala-Ala-Pro-Phe-AMC and the selective cathepsin G inhibitor I (Calbiochem) for cathepsin G activity, Suc-Ala-Ala-Pro-Val-AMC and Suc-Ala-Ala-Ala-AMC for elastase-like activity and H-Pro-Phe-Arg-AMC for kallikrein-like activity.

### 2.9 Azocasein Assay in Acute Colitis Rats

Fecal supernatants (10 μl) were incubated with 70 μl reaction buffer (0.15 M NaCl, 20 mM Tris-HCl, pH 8.3) and 70 μl azocasein (0.5%) for 20 min at 40°C. Subsequently, the reaction was stopped by adding 70 μl trichloroacetic acid (10%). The samples were then centrifuged at 4000 rpm during 2 min and the absorption of the supernatant was measured on a spectrophotometer at 340 nm. Enzymatic activities were normalized to protein content, assessed with Pierce™ BCA Protein Assay Kit (ThermoFisher Scientific, Rockford, IL, United States).

### 2.10 Statistical Analysis

All data are presented as mean ± SEM or as median (IQR), depending on asymmetry of the underlying distribution of the variable. The statistical analysis was performed using SPSS Statistics version 24.0 (IBM). Data were analyzed using a Generalized Estimating Equations (GEE) approach for the continuous endpoint VMR, to account for association in measurements from the same animal, with the mean VMR adjusted for compound and dose. Moreover, pairwise comparisons of group-specific means were performed using the post-hoc Least Significant Difference (LSD) test. Alternatively, a Bonferroni-Holm correction was performed to correct for multiple testing and control for the family-wise error rate across model-specific analyses. Inflammatory parameters and immunohistochemistry results were analyzed using Kruskal-Wallis test with pairwise comparisons and Bonferroni correction for multiple testing. qPCR results were analyzed using an unpaired Student’s t-test and nonparametric Mann-Whitney U tests. Proteolytic activities were analyzed with a two-way ANOVA and nonparametric Mann–Whitney U tests with Bonferroni correction for multiplicity. Fecal proteolytic activities assessed with an azocasein assay were analyzed with a Wilcoxon signed rank test. A (corrected two-sided) *p*-value < 0.05 was considered statistically significant. A supplementary chart with all the test statistics concerning normality and homoscedasticity, as well as the actual *p*-values can be found in Supplementary Material 1. Graphs were made with GraphPad Prism 7.0.

### 2.11 Active Compounds, Other Chemicals and Reagents

#### 2.11.1 Active Compounds

Nafamostat mesylate (Selleckchem^∗^, also named FUT-175) is a broad-spectrum serine protease inhibitor, commercially available in Japan for the treatment of acute pancreatitis and disseminated intravascular coagulation (Isozaki et al., 2006). The efficacy is also suggested for the prevention of post-endoscopic retrograde cholangiopancreatography (ERCP) pancreatitis (Yu et al., 2015) and it shows promising results for the treatment and/or prevention of COVID-19 (Li et al., 2021).

UAMC-00050 is a serine protease inhibitor with a well-defined multi-target inhibition profile and the following chemical structure (benzyl (1-(bis(4-acetamidophenoxy)phosphoryl)-2-(4-guanidinophenyl)ethyl)carbamate hydrochloride). This compound was developed by the Laboratory of Medicinal Chemistry of the University of Antwerp, originally patented under WO2007045496 (Augustyns et al., 2007; Joossens et al., 2007) and recently also patented for their use in PAR-related diseases under WO2017198753 (Augustyns et al., 2017).

For the inhibition profiles (displayed as IC_50_ values) of both serine protease inhibitors used in this study, we would like to refer the reader to previous publications from our research group by Ceuleers et al., (2018) and Hanning et al., (2021))

Nafamostat mesylate (0.2 mg/kg for mice; 0.1–1–10 mg/kg for rats) was dissolved in sterile water, whereas UAMC-00050 (2 mg/kg for mice; 0.1–1 mg/kg for rats) was dissolved in dimethyl sulfoxide (DMSO) (1% for mice and 5% for rats) for i. p. administration.

#### 2.11.2 Other Chemicals and Reagents

Mouse mast cell tryptase monoclonal antibody was purchased from Abcam (Cambridge, United Kingdom). Eosin and 100% ethanol were purchased from Acros Organics (Geel, Belgium). Fluorogenic substrates Boc-Gln-Ala-Arg-AMC, n-Tosyl-Gly-Pro-Arg-AMC, Suc-Ala-Ala-Pro-Phe-AMC, Suc-Ala-Ala-Pro-Val-AMC, Suc-Ala-Ala-Ala-AMC, and H-Pro-Phe-Arg-AMC were all purchased from Bachem (Bubendorf, Switzerland). Isoflurane (Furane^∗^) was purchased from Baxter (Deerfield, IL, United States). Xylazine (Rompun^∗^) was purchased from Bayer (Bayer, Leverkusen, Germany). 0.9% sodium chloride solution was purchased from Braun (Diegem, Belgium). Pentobarbital 60 mg/ml (Nembutal^∗^) was purchased from Ceva (Brussels, Belgium). Acetic acid, dimethylsulfoxide (DMSO), formaldehyde and hematoxylin, isopropanol, potassium dihydrogen phosphate, potassium hydrogen phosphate were purchased from Merck (Darmstadt, Germany). Ketamine (Ketalar^∗^) was purchased from Pfizer (Puurs, Belgium). Octylglucoside was purchased from Roth (Karlsruhe, Germany). Aminoethylcarbazole (AEC), cathepsin G inhibitor I, heparin, hexadecyltrimethylammonium hydrochloride (HTAB), hydrogen peroxide, o-dianisidine hydrochloride, trichloroacetic acid (TCA) and 2,4,6-trinitrobenzenesulphonic acid (TNBS) were purchased from Sigma-Aldrich (Sigma-Aldrich, Saint Louis, MO, United States). Goat anti-mouse IgG antibody, rat serum and Vectastain^∗^ avidin-biotin complex were obtained from Vector Laboratories (Vector Laboratories, CA, United States). The Krebs-Ringer solution had the following composition: 118.3 mM NaCl, 4.7 mM KCl, 1.2 mM MgSO_4_, 1.2 mM KH_2_PO_4_, 2.5 mM CaCl_2_, 2 mM NaCHO_3_, 0.026 mM CaEDTA and 11.1 mM glucose.

## 3 Results

### 3.1 Serine Protease Inhibitors Decrease Visceral Hypersensitivity in an Acute TNBS-Induced Colitis Rat Model

The average VMR of the vehicle-treated acute colitis rats was significantly different from the average VMR of the vehicle-treated controls, with higher values observed in the acute colitis rats, thereby indicating the presence of acute inflammatory visceral hypersensitivity characterized by visceral allodynia (10–30 mmHg) and hyperalgesia (40–60 mmHg) (adjusted Bonferroni-Holm *p*-value = 0.040, Figure 1C). A single i.p. administration of 0.1 mg/kg UAMC-00050 had no significant effect on the average VMR of the acute colitis rats (adjusted Bonferroni-Holm *p*-value = 1.000, Figure 1C). However, after administering 1 mg/kg UAMC-00050 i. p. in animals with acute colitis, a decrease in mean VMR has been observed (adjusted Bonferroni-Holm *p*-value = 0.001, Figure 1D). The dose of 1 mg/kg i. p UAMC-00050—effective in the colitis rats—had no significant effect on the sensitivity of control animals (adjusted Bonferroni-Holm *p*-value = 1.000, Figure 1E).

**FIGURE 2:**
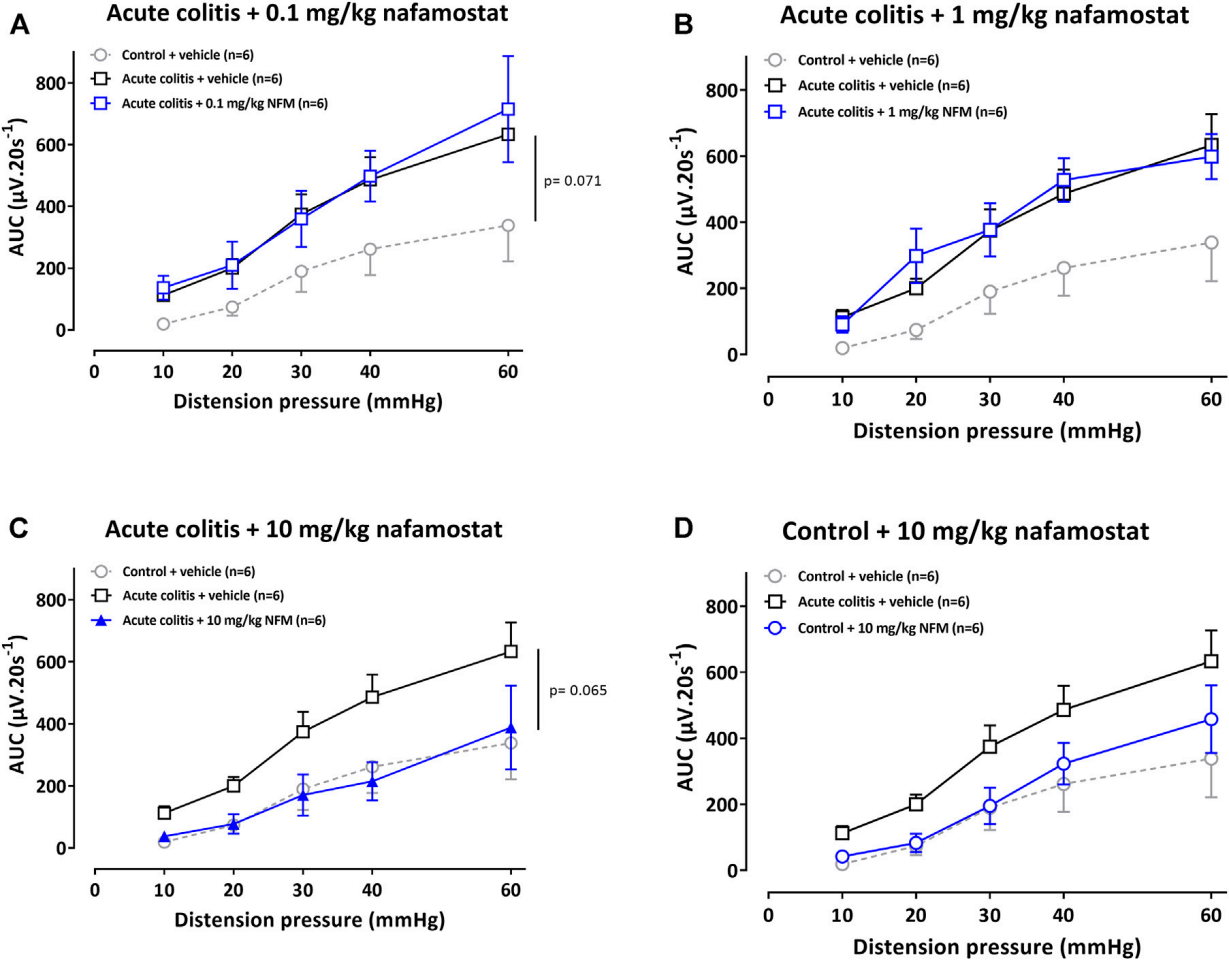
The effect of nafamostat (0.1–1–10 mg/kg) and vehicle (5% DMSO) on VMRs in acute colitis **(A–C)** and control **(D)** rats. The statistical analysis was performed on the complete dataset, but separate graphs were made for the purpose of clarification. Data are presented as mean ± SEM. Generalized Estimating Equations (GEE) + Bonferroni-Holm correction to correct for multiple testing and control for the family-wise error rate across model-specific analyses. n = 6/group; *p <0.05; significantly different from control + vehicle. #*p* <0.05; significantly different from acute colitis + vehicle.

To corroborate these findings with the trypsin-like serine protease inhibitor UAMC-00050, we also studied the effect of the broad spectrum serine protease inhibitor nafamostat mesylate in this rat model for IBD. Vehicle-treated acute colitis rats showed higher mean VMR values to colorectal distension compared to vehicle-treated controls (adjusted Bonferroni-Holm *p*-value = 0.071, Figure 2A), indicating the presence of acute inflammatory visceral hypersensitivity. A single intraperitoneal administration of 0.1 or 1 mg/kg nafamostat did not affect visceral sensitivity in acute TNBS-colitis rats (adjusted Bonferroni-Holm *p*-values = 1.000 and 1.000, Figure 2A,B) but 10 mg/kg nafamostat decreased visceral hypersensitivity in acute TNBS-colitis rats (adjusted Bonferroni-Holm *p*-value = 0.065, Figure 2C). In a dose of 10 mg/kg, nafamostat did not significantly affect visceral sensitivity in control rats (adjusted Bonferroni-Holm *p*-value = 1.000, Figure 2D).

Colonic compliance was assessed and showed a significant effect of the acute inflammation per se but not of the compounds administered (data not shown).

### 3.2 Serine Protease Inhibitors Decrease Visceral Hypersensitivity in a Neonatal Acetic Acid Mouse Model

**FIGURE 3:**
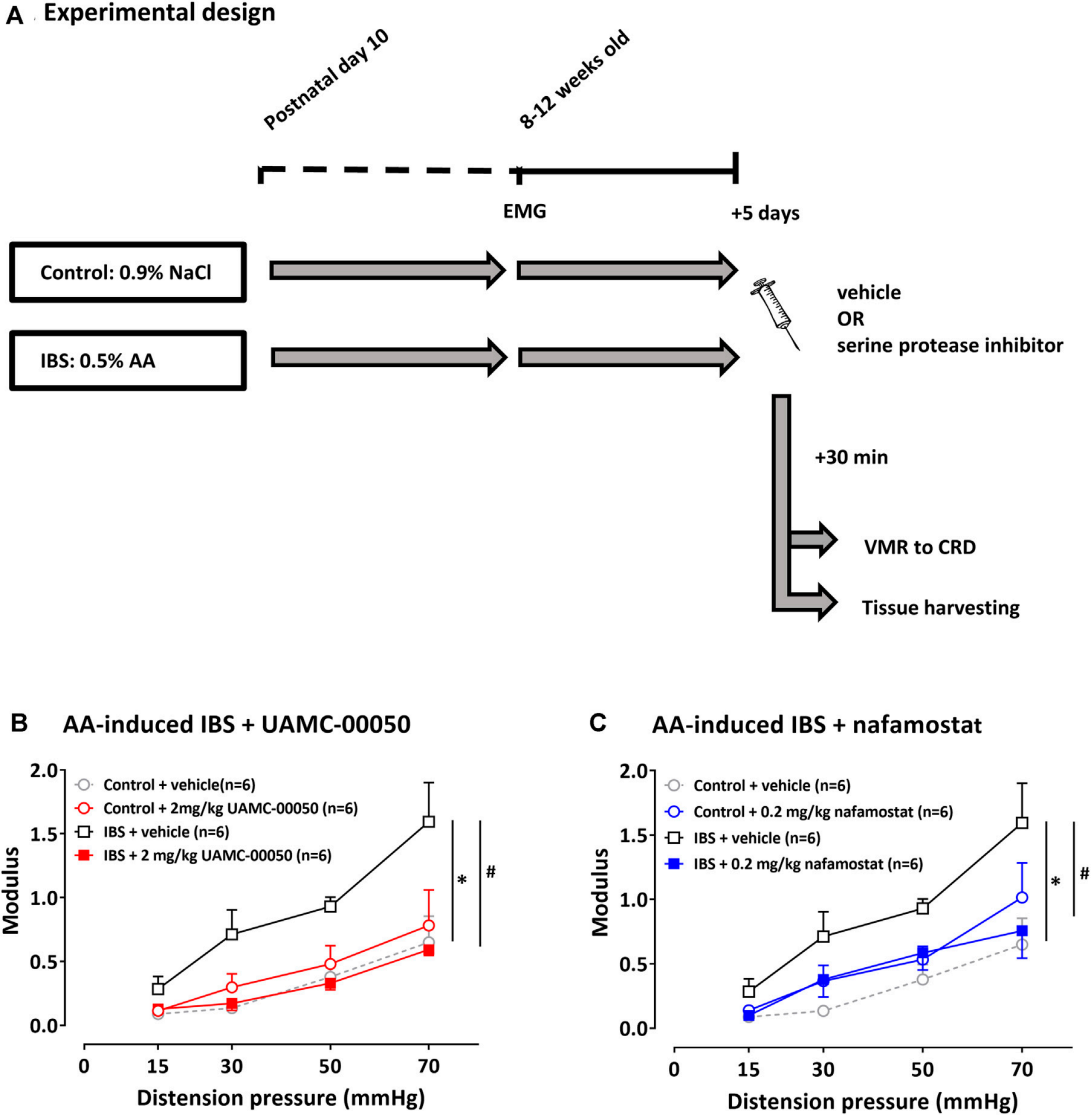
The effect of UAMC-00050 (2 mg/kg), nafamostat mesylate (0.2 mg/kg) and vehicle (1% DMSO) on VMRs in AA-induced IBS and control mice. **(A)** Overview of the experimental design in acetic acid-induced IBS mice. On postnatal day 10, mice received an intrarectal administration with AA (IBS) or 0.9% NaCl (control). In their adulthood (8–12 weeks old), EMG electrodes were implanted and 5 days later visceral sensitivity was assessed by VMR to CRD after a single i. p. injection with serine protease inhibitor/vehicle. AA: acetic acid; CRD: colorectal distension; EMG: electromyographic; IBS: irritable bowel syndrome; VMR; visceromotor response. **(B)** The effect of UAMC-00050 on VMRs in AA-induced IBS and control mice. **(C)** The effect of nafamostat mesylate (0.2 mg/kg) on VMRs in AA-induced IBS and control mice. Data are presented as mean ± SEM. Generalized Estimating Equations (GEE) and pairwise comparisons of group-specific means were performed using the LSD post-hoc test; n = 6/group; *p <0.05; significantly different from control + vehicle. #*p* <0.05; significantly different from IBS + vehicle.

Evidence for the presence of visceral hypersensitivity in mice with acetic acid-induced IBS was found by means of the significantly different average VMR with mean VMR values being higher in this group at all distension pressures (15–70 mmHg), compared to mean VMR values in control mice (LSD-adjusted *p*-value < 0.001, Figure 3B). Furthermore, at the time of VMR measurement, no signs of inflammation were detected in control and neonatal acetic acid mice, as objectified on colonic H&E stainings and mRNA cytokine levels (IL-10, IL-12, IL-1β, IL-6, IL-8 and TNF-α) in colonic samples (see Supplementary Material 2).

A single i.p. administration with 2 mg/kg UAMC-00050 30 min prior to VMR assessment significantly decreased VMRs at all distension pressures and even completely restored sensitivity to normal values (LSD-adjusted *p*-value < 0.001, Figure 3B). UAMC-00050 (2 mg/kg i. p.) did not affect visceral sensitivity in control mice (LSD-adjusted *p*-value = 0.465, Figure 3B).

To corroborate these findings with the trypsin-like serine protease inhibitor UAMC-00050, we also studied the effect of the broad spectrum serine protease inhibitor nafamostat mesylate. A single i.p. injection with nafamostat mesylate (0.2 mg/kg) 30 min before the VMR measurement leads to lower mean VMR values at all distension pressures, being significantly different from mean values in vehicle-treated mice (LSD-adjusted *p*-value = 0.009, Figure 3C). In a dose of 0.2 mg/kg, nafamostat had no significant effect on visceral sensitivity in control mice (LSD-adjusted *p*-value = 0.145, Figure 3C).

### 3.3 Serine Protease Inhibitors did not Affect Inflammatory Parameters in an Acute TNBS-Induced Colitis Rat Model After a Single i.p. Administration

On day 3, after the enema, the average colonoscopic score of the rats that received TNBS was significantly different compared to the average score of control animals, being higher in the TNBS group, thereby indicating the presence of colitis in this group (Kruskal-Wallis pairwise comparisons *p*-values < 0.05, Table 2 and Table 3). The inflammatory status of the animals was confirmed by the other post-mortem markers (macroscopy, histology and MPO activity) (Kruskal-Wallis pairwise comparisons *p*-values < 0.05, Table 2 and Table 3).

**TABLE 2:** Inflammatory parameters of all animals included in the acute TNBS colitis + UAMC-00050 experiment.

Group	Drug	N	Colonoscopy	Macroscopy	Microscopy	MPO activity
Control	Vehicle	6	0.0 (0.0)	0.0 (0.0)	0.0 (2.0)	0.8 (0.5)
	1 mg/kg	6	0.0 (0.0)	0.0 (0.0)	0.0 (0.0)	0.8 (0.3)
Acute colitis	Vehicle	6	8.0 (3.0) *#	3.5 (1.0) *#	5.5 (5.0) *#	8.4 (50.2) *#
	0.1 mg/kg	6	8.0 (3.0) *#	4.0 (1.0) *#	7.5 (2.0) *#	30.6 (36.7) *#
	1 mg/kg	6	8.5 (2.0) *#	4.0 (1.0) *#	7.0 (4.0) *#	29.3 (34.9) *#

Data are presented as median with interquartile range (IQR). Vehicle = 5% DMSO in sterile water. Non-parametric testing with Kruskal-Wallis test and pairwise comparisons with Bonferroni correction for multiple testing. *p <0.05; significantly different from control + vehicle; #*p* <0.05; significantly different from control +1 mg/kg. MPO, myeloperoxidase; N, number.

**TABLE 3:** Inflammatory parameters of all animals included in the acute TNBS colitis + nafamostat experiment.

Group	Drug	N	Colonoscopy	Macroscopy	Microscopy	MPO activity
Control	Vehicle	6	0.0 (0.0)	0.0 (0.0)	0.0 (2.0)	0.8 (0.5)
	10 mg/kg	6	0.0 (0.0)	0.0 (0.0)	0.0 (1.0)	1.1 (0.9)
Acute colitis	Vehicle	6	8.0 (3.0) *#	3.5 (1.0) *#	5.5 (5.0) *#	8.4 (50.2) *#
	0.1 mg/kg	6	6.0 (4.0) *#	3.0 (1.0) *#	8.0 (4.0) *#	22.9 (33.4) *#
	1 mg/kg	6	6.5 (2.0) *#	3.5 (2.0) *#	7.0 (3.0) *#	7.0 (17.2) *#
	10 mg/kg	6	7.0 (5.0) *#	3.5 (3.0) *#	7.0 (5.0) *#	14.4 (30.0) *#

Data are presented as median with interquartile range (IQR). Vehicle = 5% DMSO in sterile water. Non-parametric testing with Kruskal-Wallis test and pairwise comparisons with Bonferroni correction for multiple testing. *p <0.05; significantly different from control + vehicle; #*p* <0.05; significantly different from control +10 mg/kg. MPO, myeloperoxidase; N, number.

Administration with UAMC-00050 (0.1–1 mg/kg) (Kruskal-Wallis pairwise comparisons *p*-values > 0.05, Table 2) or nafamostat (0.1–1–10 mg/kg) (Kruskal-Wallis pairwise comparisons *p*-values > 0.05, Table 3) did not affect colonoscopic, macroscopic, histological nor MPO activity scores.

### 3.4 The Serine Protease Expression Profile was Altered in an Acute TNBS-Induced Colitis Rat Model

#### 3.4.1 Serine Protease mRNA Expression Levels are Altered During Acute TNBS-Induced Colitis

The mRNA expression level of a selected panel of serine proteases was determined in colonic samples from acute TNBS colitis vs. control rats (Table 4). The relative mRNA expression of matriptase (St14) in the colon of acute colitis rats was significantly downregulated compared to controls (independent samples *t*-test *p* value = 0.001, Table 4). The expression of tryptase (Tpsab1), urokinase plasminogen activator (Plau) and kallikrein 8 (KLK8) were comparable between control and acute colitis groups (Mann-Whitney U two-sided *p*-value = 0.834 for Tbsap1 and 0.248 for KLK8, independent samples *t*-test *p*-value for uPA = 1.000, Table 4). Cathepsin G, kallikrein 2 (KLK2) and kallikrein 4 (KLK4) were below detection limit in both groups (Table 4).

**TABLE 4:** Relative mRNA expression of serine proteases in colon samples in acute TNBS colitis rats.

Gene	Control	Acute colitis
Tryptase αβ1	1.30 (1.01)	1.10 (4.03)
Matriptase	1.04 (0.45)	0.53 (0.24)*
Cathepsin G	<LOD	<LOD
UPA	1.02 (0.68)	0.94 (0.88)
Kallikrein 2	<LOD	<LOD
Kallikrein 4	<LOD	<LOD
Kallikrein 8	0.84 (1.00)	0.40 (2.21)
	n = 8	n = 8

Data are expressed as relative mRNA, expression (using household genes GAPDH, and β-actin) and presented as median with interquartile range (IQR) for n = 8. Unpaired *t*-test for matriptase and uPA, and Mann-Whitney *U* test for tryptase αβ1 and kallikrein 8. Significant effect of the factor “group”; *p <0.05. LOD, limit of detection.

#### 3.4.2 Mast Cell Tryptase-Positive Cells Are Not Altered During Acute TNBS-Induced Colitis

**FIGURE 4:**
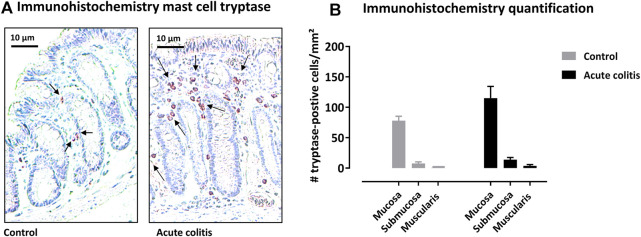
Immunohistochemistry with mast cell tryptase antibody in rat colon. **(A)** Representative images of the colonic mucosa of a control and an acute colitis animal with mast cell tryptase (purple-red). **(B)** The number of tryptase-positive mast cells per mm2 in the mucosa, submucosa and muscularis externa in control (grey bars) and acute colitis (black bars) animals. Non-parametric testing with Kruskal-Wallis test and pairwise comparisons with Bonferroni correction for multiple testing.

Mast cell tryptase was quantified in the colon of the rats using immunohistochemistry. In the colon of control rats, the following level of distribution of mast cell tryptase could be observed: mucosa > submucosa > muscularis externa (Figure 4B). In acute TNBS-colitis animals, the distribution of tryptase positive cells was similar. The total number of mast cell tryptase positive cells was not significantly altered in acute colitis rats compared to control animals in the different layers (Kruskal-Wallis pairwise comparisons *p*-values = 0.616 for mucosa, 0.479 for submucosa and 0.312 for muscularis, Figure 4B).

### 3.5 Serine Protease Inhibition Altered Proteolytic Activities in an Acute TNBS-Induced Colitis Rat Model

**FIGURE 5:**
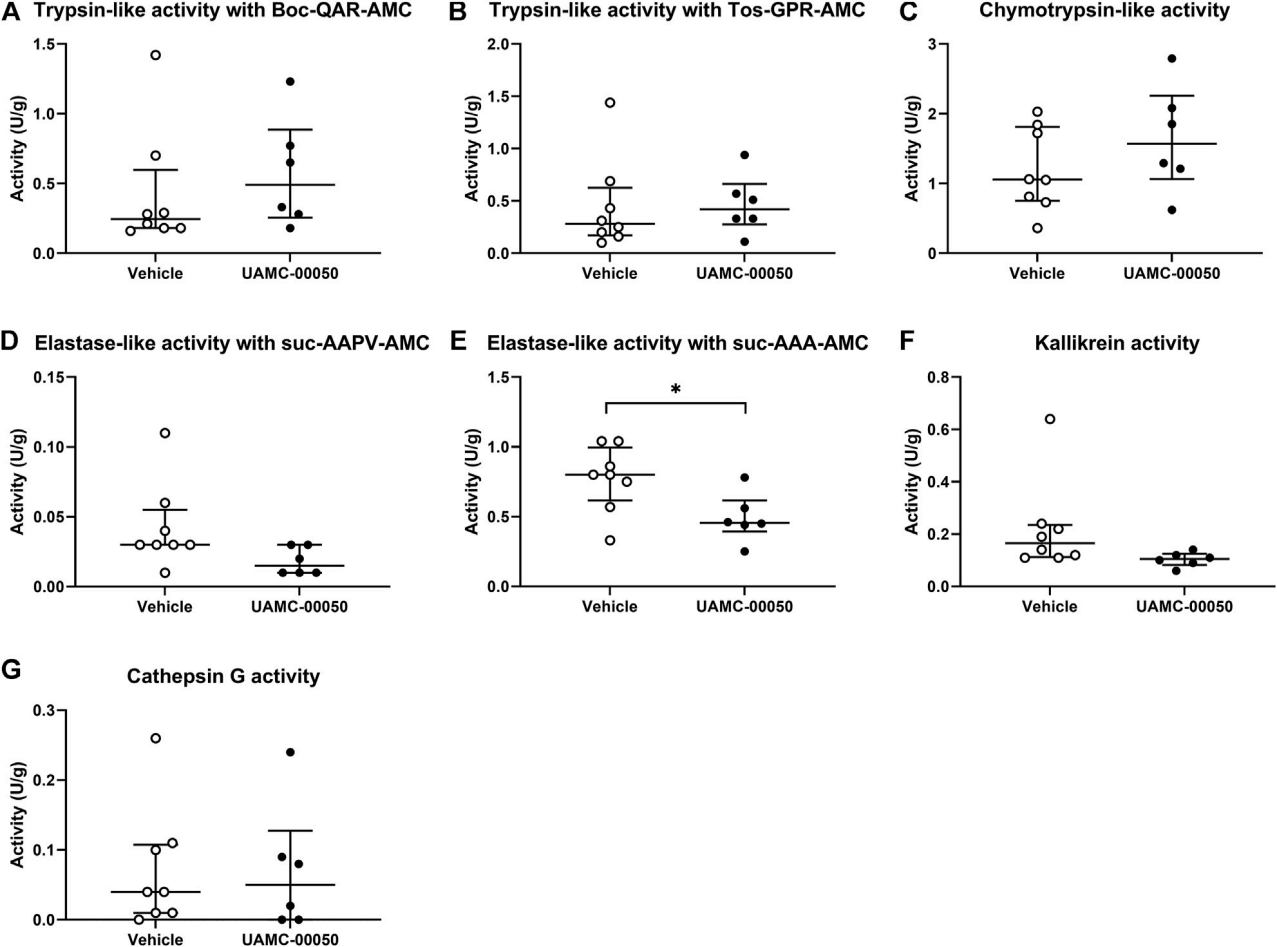
Proteolytic activities measured with fluorogenic substrates in colon samples from acute colitis rats treated with UAMC-00050 (1 mg/kg) or vehicle (5% DMSO). **(A)** Trypsin-like activity measured with Boc-QAR-AMC **(B)** Trypsin-like activity measured with Tos-GPR-AMC **(C)** Chymotrypsin-like activity measured with suc-AAPF-AMC **(D)** Elastase-like activity measured with Suc-AAPV-AMC **(E)** Elastase-like activity measured with Suc-AAA-AMC **(F)** Kallikrein activity measured with H-PFR-AMC **(G)** Cathepsin-G activity measured with suc-AAPF-AMC and the cathepsin G inhibitor I. Data are presented as median with interquartile range (IQR). Data were analyzed by Mann–Whitney U tests with Bonferroni correction for multiplicity; n = 6–8 per group. Analysis was performed on the full dataset (involving the four combinations of control/post-colitis and vehicle/UAMC-00050), however only data from the acute colitis group were shown in this figure since data from the other groups were previously published. *p < 0.05; significantly different from colitis + vehicle.

We measured no significant differences in proteolytic activities in colonic samples between vehicle-treated acute colitis and control animals for trypsin-like, chymotrypsin-like, elastase-like, cathepsin G and kallikrein-like activities (De bruyn et al., 2021). Interestingly, the administration of UAMC-00050 resulted in a decreased elastase-like activity measured with Suc-Ala-Ala-Ala-AMC in the colon of acute colitis rats (Mann-Whitney U two-sided *p*-value = 0.028, Figure 5). No significant differences were observed between treated and untreated acute colitis animals for trypsin-like, chymotrypsin-like, cathepsin G and kallikrein activities in colon samples (Mann-Whitney U two-sided *p*-values = 0.245 for TL-Boc, 0.439 for TL-Tos, 0.197 for chymotrypsin-like, 0.697 for cathepsin G and 0.090 for KLK, Figure 5).

**FIGURE 6:**
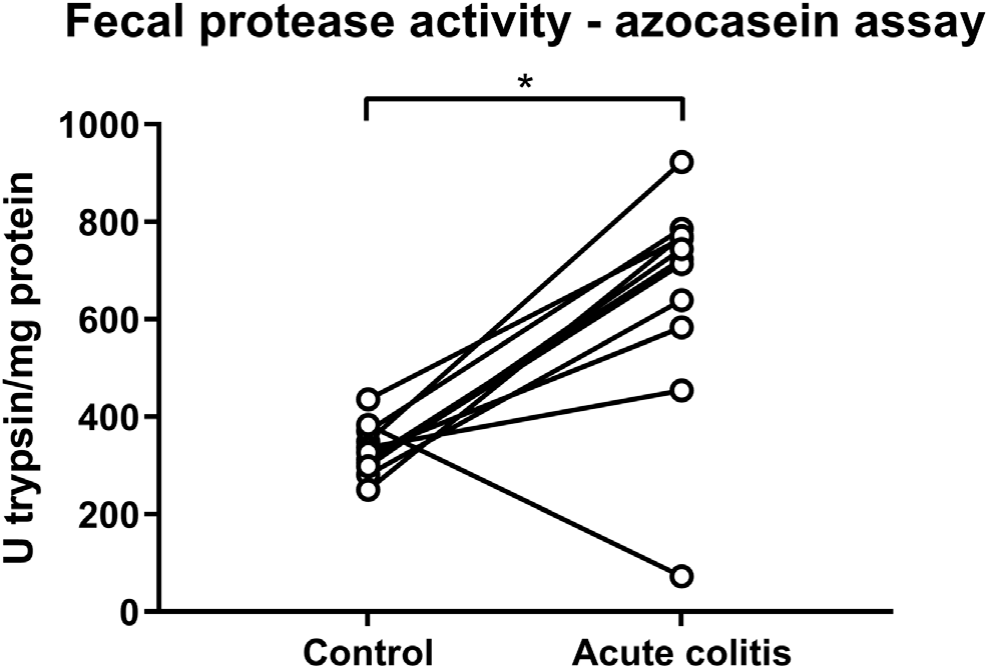
Fecal protease activity in control vs. acute colitis rats as measured with the azocasein assay. The protease activity expressed as mean U trypsin/mg protein ± SEM in fecal samples of control and acute colitis animals. Wilcoxon signed rank test; n = 12; *p* <0.05; significant effect of the factor “group”.

**FIGURE 7:**
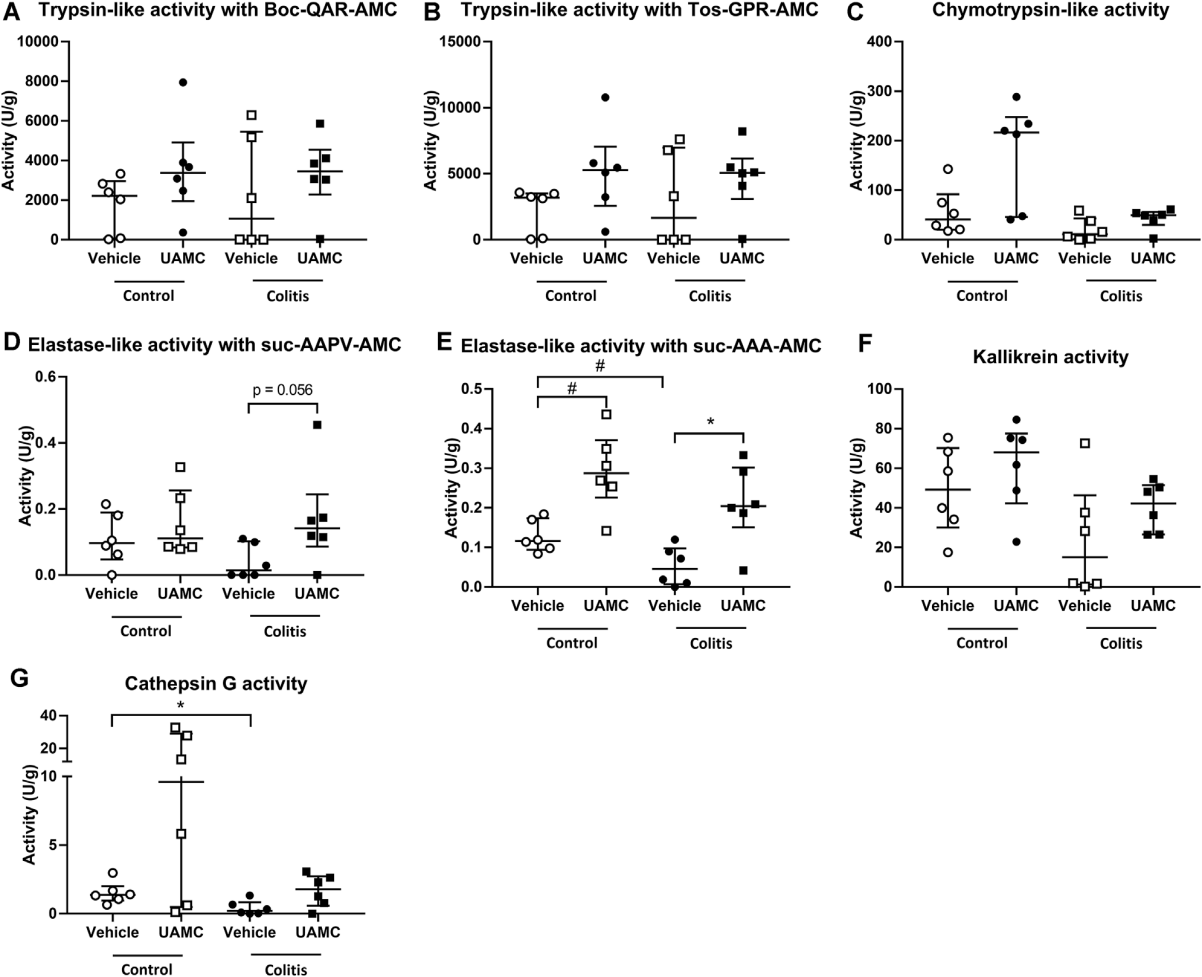
Proteolytic activities measured with fluorogenic substrates in fecal samples from control and acute colitis rats treated with UAMC-00050 (1 mg/kg) or vehicle (5% DMSO). **(A)** Trypsin-like activity measured with Boc-QAR-AMC **(B)** Trypsin-like activity measured with Tos-GPR-AMC **(C)** Chymotrypsin-like activity measured with suc-AAPF-AMC **(D)** Elastase-like activity measured with Suc-AAPV-AMC **(E)** Elastase-like activity measured with Suc-AAA-AMC **(F)** Kallikrein activity measured with H-PFR-AMC **(G)** Cathepsin-G activity measured with suc-AAPF-AMC and the cathepsin G inhibitor I. Data are presented as median with interquartile range (IQR). Data were analyzed using two-way ANOVA with “treatment” and “group” factors followed by unpaired T-tests with Bonferroni correction for multiplicity (for trypsin-like activity measured with Boc-QAR-AMC and Tos-GPR-AMC, elastase-like activity measured with Suc-AAA-AMC and kallikrein activity), and by Mann–Whitney U tests with Bonferroni correction for multiplicity (for chymotrypsin-like activity, elastase-like activity measured with suc-AAPV-AMC and cathepsin-G activity); n = 6–8 per group. *p < 0.05; significantly different from colitis + vehicle; #*p* < 0.05; significantly different from control + vehicle.

On the contrary, total protease activity assessed by means of an azocasein assay was significantly increased in fecal samples of acute colitis rats compared to controls (Wilcoxon signed-rank *p*-value = 0.006, Figure 6). Moreover, fecal samples from vehicle-treated acute colitis animals displayed a significantly lower elastase-like activity measured with Suc-Ala-Ala-Ala-AMC (Bonferroni-corrected unpaired *t*-test *p*-value = 0.014, Figure 7E) and cathepsin G activity (Mann-Whitney U two-sided *p*-value = 0.032, Figure 7G) compared to vehicle-treated controls. Besides, treatment with UAMC-00050 significantly increased elastase-like activity measured with Suc-Ala-Ala-Ala-AMC (Bonferroni-corrected unpaired *t*-test *p*-value = 0.006, Figure 7E) and showed a trend towards an increase in elastase-like activity measured with Suc-Ala-Ala-Pro-Val-AMC in fecal samples from acute colitis rats (Mann-Whitney U two-sided *p*-value = 0.056, Figure 7D).

## 4 Discussion

In this study, we provide new evidence for serine protease inhibitors such as UAMC-00050 and nafamostat as promising therapeutic agents for the treatment of visceral hypersensitivity. We show that UAMC-00050 and nafamostat potently reduce visceral pain in a mouse model for IBS as well as in a rat model for IBD.

We have previously demonstrated that serine protease inhibition with UAMC-00050 showed potent therapeutic efficacy in a post-inflammatory rat model for IBS after intraperitoneal and intracolonic administration (Ceuleers et al., 2018; Hanning et al., 2021). To further validate UAMC-00050 as a promising compound for the treatment of visceral pain, we here substantiated our findings in a neonatal acetic acid-induced mouse model as an additional preclinical animal model with a different onset for IBS and in a different species. Our results show that visceral sensitivity was significantly enhanced in acetic acid-induced IBS mice and that a single intraperitoneal administration of UAMC-00050 (2 mg/kg) completely reversed visceral hypersensitivity to control values. Administration of UAMC-00050 was without effect in controls. Moreover, we also demonstrated a similar decrease in visceral pain in acetic acid-induced IBS mice after a single intraperitoneal administration of the broad spectrum protease inhibitor nafamostat mesylate (0.2 mg/kg). When taking into account the dose conversion from rats to mice (x2), our results with UAMC-00050 are completely in line with our previous findings: a complete restoration of visceral sensitivity after a single i. p. injection with 1 mg/kg UAMC-00050 in a TNBS-induced post-inflammatory rat model for IBS (Ceuleers et al., 2018). The comparable results of UAMC-00050 in the TNBS post-inflammatory model for IBS in rats and the neonatal acetic acid-induced model for IBS in mice strongly support UAMC-00050 as a promising compound for the treatment of visceral pain in IBS.

Because of the promising effect of UAMC-00050 that we observed in two different IBS models for visceral pain and since chronic abdominal pain is also a common symptom in IBD, we additionally investigated the efficacy of UAMC-00050 in a rat model for IBD. The TNBS model of acute colitis is a well described experimental model for an acute IBD flare, most commonly presented as a model for Crohn’s disease (Morris et al., 1989). We found that TNBS-induced acute colitis in rats significantly enhanced visceral pain, characterized by visceral allodynia and hyperalgesia, in line with previously reported results from our research group (Vermeulen et al., 2013). UAMC-00050 reduced this visceral hypersensitivity without affecting visceral pain in controls.

An interesting finding of our study is that UAMC-00050 reduced both visceral allodynia (10–30 mmHg) and hyperalgesia (40–60 mmHg) in the two different animal models of visceral pain. These findings are completely in line with a study from Auge et al., (2009) where they demonstrated a state of visceral hypersensitivity, characterized by visceral allodynia as well as hyperalgesia, after the intracolonic administration of a PAR2-agonist (SLIGRL-NH_2_) in male C57Bl6 mice, thereby emphasizing the role of serine proteases in both allodynia and hyperalgesia. Our results are in contrast with a study from Moussa et al., (2012) who found an effect on hyperalgesia, but not on allodynia. In that study, female Wistar rats received a daily oral administration with a fermented soy germ (FSG) extract containing isoflavones and a Bowman-Birk inhibitor (trypsin-like serine protease inhibitor) for 15 days. On day 15, acute TNBS-colitis was induced and 5 days later, visceral sensitivity was measured. The vehicle-treated acute colitis rats presented increased VMRs at 30–45–60 mmHg, demonstrating the presence of visceral hypersensitivity. The rats treated with FSG, showed a significant reduction in VMR, but this effect was only seen at the higher pressures (45–60 mmHg). In contrast to our acute colitis rats, displaying a state of hypersensitivity, Annahazi et al., (2009) found that mice presented hyposensitivity after the infusion of colonic supernatant from UC patients. This discrepancy could possibly be explained by the fact that an acute TNBS colitis rodent model shares more characteristics with human CD than with UC (Antoniou et al., 2016) and that potentially other serine proteases and/or PARs are involved in our animal model compared to the model that was used by Annahazi et al., (2009). Additionally, after the administration of a PAR4-antagonist the mice were hypersensitive, while a mixture of aprotinin and soybean trypsin inhibitor (SBTI) could restore the sensitivity to normal levels (Annahazi et al., 2009).

We compared the effect of UAMC-00050 with that of the broad spectrum serine protease inhibitor nafamostat and found that nafamostat reduced visceral hypersensitivity in our rat model for IBD without affecting visceral pain in controls. So far, nafamostat was mostly studied related to visceral pain only in IBS models using supernatant of IBS patients in mouse models (Cenac et al., 2007; Wang et al., 2015). Moreover, research on the effect of serine protease inhibitors in experimental animal models for IBD have mainly focused on their effect on inflammation. Therefore, to our knowledge, we are the first to consider the effects of serine protease inhibition in a therapeutic setting on visceral pain in an acute animal model for IBD. Regarding the effect on inflammation, a decrease in inflammation has indeed been shown in both DSS colitis mice (Cho et al., 2011) as well as in TNBS colitis rats (Isozaki et al., 2006) after a 6 day oral and a 6 day intracolonic treatment with nafamostat respectively. In our experiments, serine protease inhibition with UAMC-00050 or nafamostat mesylate did not affect the parameters scoring the intestinal inflammation such as endoscopy, macroscopy, microscopy and MPO activity, which was also not expected because animals were treated for only 30 min with the compound before assessing the VMR. This is in line with the results observed after an intraperitoneal administration with UAMC-00050 in a post-inflammatory TNBS rat model (Ceuleers et al., 2018). However, we previously demonstrated an increased MPO activity after local colonic administration of UAMC-00050. The explanation for this increase is not entirely clear but might be explained by 1) the disturbance of the protease–anti-protease balance interfering with inflammatory or anti-inflammatory mediators, 2) an interaction with other luminal components (e.g., the microbiome or its metabolites), or 3) the presence of neutrophil extracellular traps, which could release proteins such as MPO but also neutrophil elastase or cathepsin G (Drury et al., 2021; Hanning et al., 2021). Interestingly, we recently demonstrated an amelioration of the inflammatory parameters after a 2-week i. p. treatment with UAMC-00050 in a chronic colitis T cell transfer mouse model (Van Spaendonk et al., 2021).

Remarkably, nafamostat decreased acute TNBS colitis-induced visceral hypersensitivity in the highest dose used (10 mg/kg), which is in contrast with previously obtained results in post-inflammatory visceral hypersensitivity where nafamostat showed its greatest potential in the lowest dose used (Ceuleers et al., 2018). We hypothesize that this difference in response to the same protease inhibitor might be attributed to a difference in protease profiles in acute TNBS colitis rats vs. post-inflammatory rats (as discussed further on).

In a second part of this study, we substantiated the serine protease profiles in the colonic wall of acute colitis rats. We first investigated the mRNA expression levels of a panel of serine proteases in colonic samples of acute colitis rats compared to controls. Consistent with previous studies, this study demonstrated a significant downregulation of mRNA of matriptase in acute TNBS colitis animals compared to controls. Decreased matriptase mRNA expression levels were also observed in DSS colitis mice, an experimental animal model for IBD (Buzza et al., 2017), as well as in inflamed colonic tissue from Crohn and ulcerative colitis patients (Netzel-Arnett et al., 2012). Secondly, we studied the presence of tryptase positive mast cells in the colon of acute colitis rats at the protein level and found no significant difference in the number of tryptase positive mast cells in the different layers of the colon of acute TNBS colitis animals compared to the controls. This observation is in accordance with our findings at the mRNA level in acute TNBS colitis animals, but in contrast with findings in the recent literature. As such, increased levels of intestinal mast cell tryptase are observed in both experimental rat and mouse models for IBD (Hamilton et al., 2011; Lohman et al., 2012). Furthermore, several clinical studies also revealed higher amounts of tryptase in colonic biopsies from IBD patients (Raithel et al., 2001; Cenac et al., 2007; Peterson et al., 2007).

Finally, we assessed the effect of UAMC-00050 on proteolytic activities using a panel of substrates in the colon and feces of acutely inflamed rats. A first interesting observation was the presence of different effects on elastase-like activity. More specifically, we determined a minor decrease in elastase-like activity measured with suc-AAA-AMC in fecal samples and an unaltered elastase-like activity measured with suc-AAA-AMC in colon samples (De bruyn et al., 2021) and with suc-AAPV-AMC in both colon (De bruyn et al., 2021) and fecal samples from untreated acute colitis vs. control rats. This is in contrast with previous studies detecting an elevated elastase activity in colonic tissue from IBD patients (Motta et al., 2012) as well as in the fecal supernatant from a subpopulation of postinfectious IBS patients with an increased proteolytic activity (Edogawa et al., 2020). A possible explanation for this discrepancy might lay in the fact that the number of samples in our study was too low to detect a significant difference. Taking a closer look at the effects of UAMC-00050, we found that the elastase-like activity was decreased in colon but increased in fecal samples from UAMC-00050-treated vs. vehicle-treated acute colitis animals. The discrepancy between the observations in colon vs. fecal samples could possibly be explained by 1) the inhibition profile where no efficient inhibition of elastase (IC_50_ neutrophil elastase >2.5 μM, IC_50_ pancreatic elastase >10 μM) could be detected *in vitro* with UAMC-00050 (Hanning et al., 2021) or 2) increased elastase activity levels produced by bacterial enzymes from e.g., *E. coli* (Patel et al., 2004) or *B. licheniformis* (Georgieva et al., 2005; Hanning et al., 2021). Interestingly, inhibition of general elastase activity by delivery of the protease inhibitor elafin had protective effects in a DSS- and TNBS-induced colitis mouse model (Motta et al., 2011; Motta et al., 2012). Taken together, this points towards an important role for elastase-like activity in IBD.

In order to explain the mechanism underlying the decreased visceral pain in acute colitis animals treated with UAMC-00050, despite the increased elastase-like and chymotrypsin-like activities we hypothesize a different role for neutrophil elastase and chymotrypsin. Indeed, it has been reported that both neutrophil elastase and chymotrypsin could have inhibitory effects on PAR2 (Bunnett et al., 2019). As a consequence, inhibition of PAR2 could result in a decrease in abdominal pain. These antinociceptive effects of PAR2 inhibition have been demonstrated previously (Cenac et al., 2007; Wang et al., 2015).

Fecal protease activity assessed by an azocasein assay was significantly increased in acute TNBS colitis rats compared to control animals. Similar differences in fecal protease activity have been documented in an acute TNBS colitis animal model for IBD (Moussa et al., 2012), in addition to UC patient samples (Roka et al., 2007; Annahazi et al., 2009). As previously reported, the azocasein assay is very non-specific and thus conclusions from data obtained with this assay must be treated with caution. Fortunately, better tools such as the fluorogenic substrates (Edgington-Mitchell, 2015; Ceuleers et al., 2018), that were also used in this study, and activity-based probes (Denadai-Souza et al., 2018) are available and allow a better characterization of proteolytic activities.

If we compare these serine protease profiles from acute TNBS colitis rats to the profiles described in our post-inflammatory rats (Ceuleers et al., 2018; Hanning et al., 2021), we can conclude that tryptase expression is significantly elevated in the post-inflammatory phase of colitis, while matriptase mRNA expression is only significantly decreased in the acute colitis phase. Besides, different proteolytic activities were elevated in colonic samples from acute vs. post-inflammatory phase; proteolytic activities were not significantly altered in acute colitis rats, while trypsin-like activity was increased in post-inflammatory rats, compared to control animals (Ceuleers et al., 2018; De bruyn et al., 2021; Hanning et al., 2021). Furthermore, an i. p. administration of UAMC-00050 only affected elastase-like and chymotrypsin-like activity in acute TNBS colitis rats, while an intracolonic administration with UAMC-00050 significantly affected trypsin-like activity in post-inflammatory animals (Hanning et al., 2021). These remarkable differences in the serine protease profile of rats in an acute vs. post-inflammatory phase, highlight a potentially important role for protease profiling of colon or fecal samples in patients with gastrointestinal diseases such as IBD or IBS to predict the possible effect of serine protease inhibitors as a potential therapeutic strategy in a specific patient.

In conclusion, our results suggest that serine proteases play an important role in visceral hypersensitivity in neonatal acetic acid mouse model and an acute TNBS colitis rat model. Inhibition of serine proteases potently reduced visceral hypersensitivity in these experimental models for IBS and IBD, providing fundamental evidence for serine protease inhibitors as a promising new therapeutic strategy for abdominal pain in gastrointestinal diseases. Besides, the profiling of the serine protease activity (e.g., trypsin-like, chymotrypsin-like, elastase-like, kallikrein and cathepsin G activities) in colonic tissue or feces might offer promising results both for an individualized approach in patient diagnosis, treatment choice and monitoring of treatment.

## Data Availability

The raw data supporting the conclusion of this article will be made available by the authors, without undue reservation.
