# Supplementary material for: The Effect of Serine Protease Inhibitors on Visceral Pain in Different Rodent Models With an Intestinal Insult
Source: Front Pharmacol. 2022 Jun 2;13:765744. doi: 10.3389/fphar.2022.765744 (PMC9201642; doi:10.3389/fphar.2022.765744)
Supplement: Supplementary file 1 [file DataSheet2.pdf]

Supplementary file 2 - Inflammatory status of the colon in neonatal acetic acid-induced mice

For microscopic evaluation of inflammation in the mouse colon, a colonic segment was fixed in 4% formaldehyde for 24h and embedded in paraffin for hematoxylin & eosin staining.

H&E stainings from control and neonatal acetic acid-induced IBS mice (n=6/group) were microscopically scored using a previously published scoring system (Heylen et al., 2013; Van Spaendonk et al., 2021). Briefly, the following parameters were scored: degree of inflammatory infiltrates (0-3), presence of goblet cells (0-1), changes in crypt architecture (0-3), presence of crypt abscesses (0-1), presence of mucosal erosion and/or ulceration (0-1) and the number of layers involved in inflammation from mucosal towards transmural involvement (0-3), resulting in a total score ranging between 0 and 13. The samples were scored in a blinded way and all parameters showed a score of 0 both in control mice and neonatal acetic acid mice. Representative images from an H&E staining of the colon of a control and IBS mouse are shown in **Figure 1**.

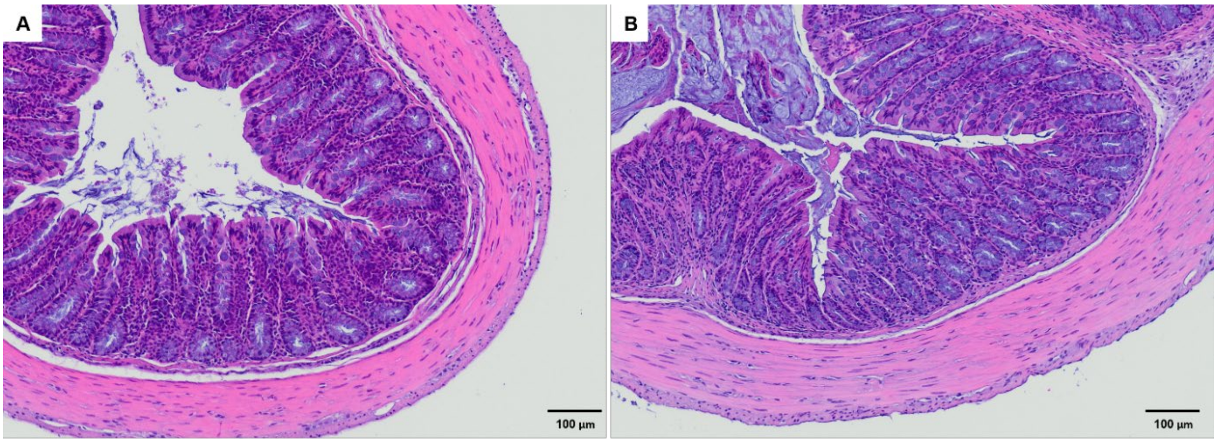

Figure 1. Representative image from an H&E staining of the colon of a control (A) and a neonatal acetic acid-induced IBS mouse (B) in their adulthood.

Next to H&E stainings, mRNA cytokine levels (IL-10, IL-12, IL1B, TNFa, , IL6, IL8,) were quantified by RT-qPCR using a previously published method (Plaeke et al., 2020) in colon samples from control vs acetic acid-induced IBS mice. The sequences of the primers as well as the housekeeping genes used (*Rpl4* and *Rps29)* are shown in **Table 1**. No significant differences were found between control and IBS mice (**Figure 2**).

Supplementary file 2 - Inflammatory status of the colon in neonatal acetic acid-induced mice

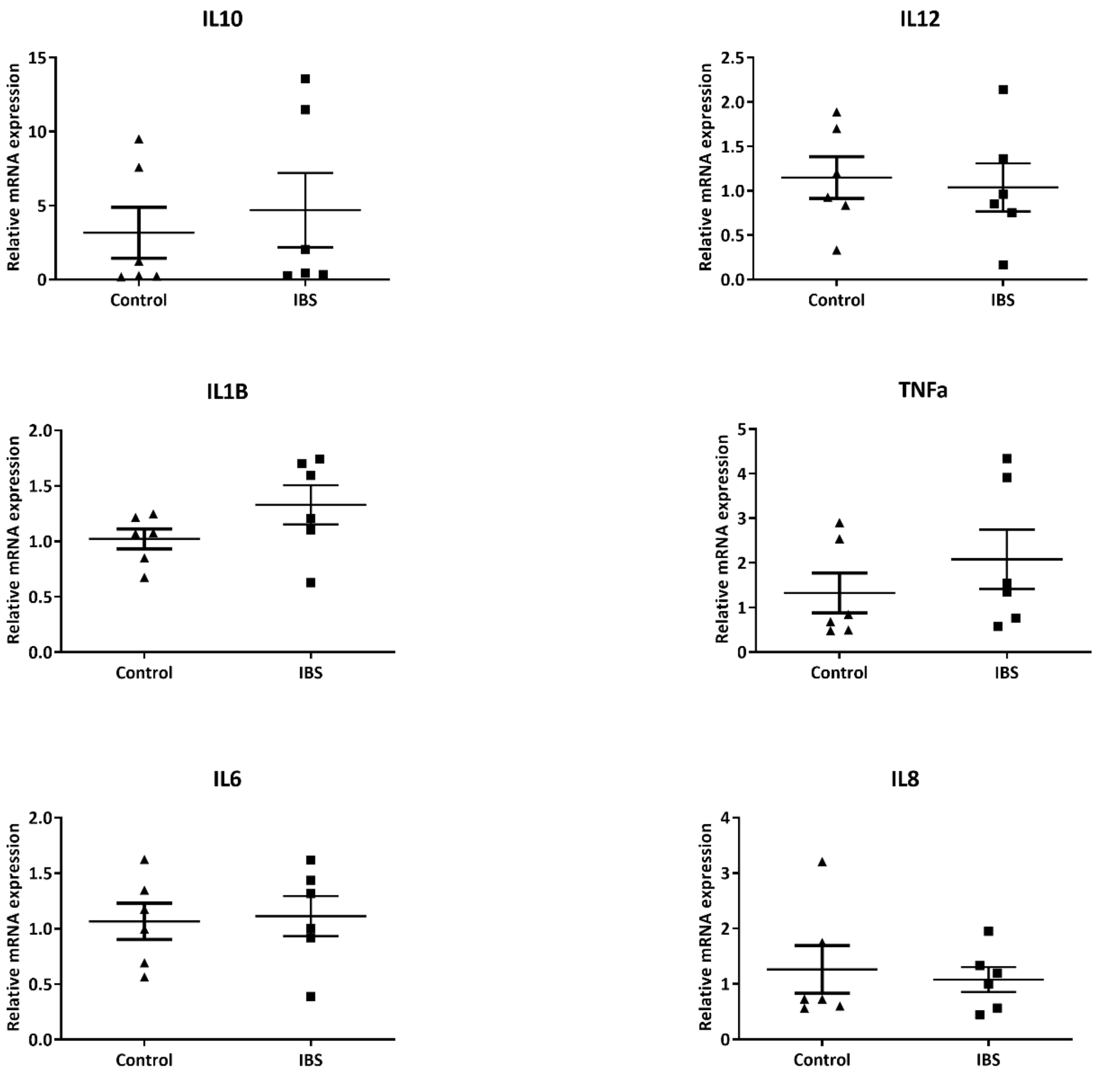

Figure 2. mRNA cytokine levels (IL-10, IL-12, IL1B, TNFa, IL6, IL8) in colon samples from control vs acetic acid-induced IBS mice. Unpaired T-test (IL-12, IL-1B, IL-6) or Mann-Whitney U test (IL-10, TNFa, IL8). No significant differences were found between control and IBS mice.

Supplementary file 2 - Inflammatory status of the colon in neonatal acetic acid-induced mice

Table 1. SYBR Green primers used for RT-qPCR analysis of mouse colonic samples

**Primer name**	**Sequence 5'-3'**
m_RPL4_FW	CCGTCCCCTCATATCGGTGTA
m_RPL4_REV	GCATAGGGCTGTCTGTTGTTTTT
m_RPS29_FW	GTCTGATCCGCAAATACGGG
m_RPS29_REV	AGCCTATGTCCTTCGCGTACT
IL-10_m_FW	GCTCTTACTGACTGGCATGAG
IL-10_m_REV	CGCAGCTCAGGAGCATGTG
IL12 _m_FW	CAATCACGCTACCTCCTCTTTT
IL12 _m_REV	CAGCAGTGCAGGAATAATGTTTC
IL1beta _m_FW	GAAATGCCACCTTTTGACAGTG
IL1beta _m_REV	TGGATGCTCTCATCAGGACAG
TNFalfa _m_FW	CAGGCGGTGCCTATGTCTC
TNFalfa _m_REV	CGATCACCCCGAAGTTCAGTAG
IL-6_m_FW	ACACATGTTCTCTGGGAAATCGTGG
IL-6_m_REV	TCTGCAAGTGCATCATCGTTGTTCA
IL8 _m_FW	TCGAGACCATTTACTGCAACAG
IL8 _m_REV	CATTGCCGGTGGAAATTCCTT

References

Heylen, M., Deleye, S., De Man, J.G., Ruyssers, N.E., Vermeulen, W., Stroobants, S., et al. (2013). Colonoscopy and µPET/CT are valid techniques to monitor inflammation in the adoptive transfer colitis model in mice. *Inflamm Bowel Dis* 19(5), 967-976. doi: 10.1097/MIB.0b013e3182802c7c.

Plaeke, P., De Man, J.G., Smet, A., Malhotra-Kumar, S., Pintelon, I., Timmermans, J.P., et al. (2020). Effects of intestinal alkaline phosphatase on intestinal barrier function in a cecal ligation and puncture (CLP)-induced mouse model for sepsis. *Neurogastroenterol Motil* 32(3), e13754. doi: 10.1111/nmo.13754.

Van Spaendonk, H., Ceuleers, H., Smet, A., Berg, M., Joossens, J., Van der Veken, P., et al. (2021). The Effect of a Novel Serine Protease Inhibitor on Inflammation and Intestinal Permeability in a Murine Colitis Transfer Model. *Frontiers in Pharmacology* 12(1601). doi: 10.3389/fphar.2021.682065.
