# Supplementary material for: The Effect of Serine Protease Inhibitors on Visceral Pain in Different Rodent Models With an Intestinal Insult
Source: Front Pharmacol. 2022 Jun 2;13:765744. doi: 10.3389/fphar.2022.765744 (PMC9201642; doi:10.3389/fphar.2022.765744)
Supplement: Supplementary file 2 [file DataSheet1.pdf]

Supplementary file 1 - Details on statistical analysis of all data included in the manuscript

1. Inflammatory parameters acute colitis rats UAMC-00050

- Data are not normally distributed (Shapiro-Wilk test).
- Equality of variances cannot be assumed (Levene's test).
- Non-parametric testing was performed on these data with a Kruskal-Wallis test and pairwise comparisons with Bonferroni correction for multiple tests.
- p-values for colonoscopy day 3

Sample 1 – Sample 2	p-value
0-4	1,000
0-2	0,005
0-1	0,003
0-3	0,000
4-2	0,005
4-1	0,003
4-3	0,000
2-1	0,905
2-3	0,484
1-3	0,561

Each row tests the null hypothesis that the Sample 1 and Sample 2 distributions are the same.
Group 0 = control + vehicle; Group 1 = TNBS + vehicle; Group 2 = TNBS + 0.1 mg/kg UAMC;
Group 3 = TNBS + 1 mg/kg UAMC; Group 4 = control + 1 mg/kg UAMC

- p-values for macroscopy

Sample 1 – Sample 2	p-value
0-4	1,000
0-1	0,011
0-2	0,001
0-3	0,000
4-1	0,011
4-2	0,001
4-3	0,000
1-2	0,429
1-3	0,268
2-3	0,752

Each row tests the null hypothesis that the Sample 1 and Sample 2 distributions are the same.
Group 0 = control + vehicle; Group 1 = TNBS + vehicle; Group 2 = TNBS + 0.1 mg/kg UAMC;
Group 3 = TNBS + 1 mg/kg UAMC; Group 4 = control + 1 mg/kg UAMC

Supplementary file 1 - Details on statistical analysis of all data included in the manuscript

- p-values for microscopy

Sample 1 – Sample 2	p-value
4-0	0,687
4-1	0,007
4-3	0,001
4-2	0,000
0-1	0,021
0-3	0,002
0-2	0,002
1-3	0,460
1-2	0,440
3-2	0,973

Each row tests the null hypothesis that the Sample 1 and Sample 2 distributions are the same.
Group 0 = control + vehicle; Group 1 = TNBS + vehicle; Group 2 = TNBS + 0.1 mg/kg UAMC;
Group 3 = TNBS + 1 mg/kg UAMC; Group 4 = control + 1 mg/kg UAMC

- p-values for MPO activity

Sample 1 – Sample 2	p-value
4-0	0,974
4-1	0,010
4-2	0,007
4-3	0,004
0-1	0,011
0-2	0,008
0-3	0,004
1-2	0,922
1-3	0,768
2-3	0,844

Each row tests the null hypothesis that the Sample 1 and Sample 2 distributions are the same.
Group 0 = control + vehicle; Group 1 = TNBS + vehicle; Group 2 = TNBS + 0.1 mg/kg UAMC;
Group 3 = TNBS + 1 mg/kg UAMC; Group 4 = control + 1 mg/kg UAMC

2. Inflammatory parameters acute colitis rats nafamostat

- Data are not normally distributed (Shapiro-Wilk test).
- Equality of variances cannot be assumed for colonoscopy, macroscopy and MPO activity. Equality of variances can be assumed for microscopy (Levene's test).
- Non-parametric testing was performed on these data with a Kruskal-Wallis test and pairwise comparisons with Bonferroni correction for multiple tests.
- p-values for colonoscopy day 3

Sample 1 – Sample 2	p-value
1-6	1,000
1-5	0,006
1-3	0,004
1-4	0,003
1-2	0,000
6-5	0,006
6-3	0,004
6-4	0,003
6-2	0,000
5-3	0,933
5-4	0,833
5-2	0,441
3-4	0,900
3-2	0,492
4-2	0,575

Each row tests the null hypothesis that the Sample 1 and Sample 2 distributions are the same.
Group 1 = control + vehicle; Group 2 = TNBS + vehicle; Group 3 = TNBS + 0.1 mg/kg nafamostat;
Group 4 = TNBS + 1 mg/kg nafamostat; Group 5 = TNBS + 10 mg/kg nafamostat; Group 6 = Control + 10 mg/kg nafamostat

- p-values for macroscopy

Sample 1 – Sample 2	p-value
1-6	1,000
1-3	0,006
1-2	0,002
1-4	0,001
1-5	0,001
6-3	0,006
6-2	0,002
6-4	0,001
6-5	0,001
3-2	0,762
3-4	0,624
3-5	0,555
2-4	0,852
2-5	0,773
4-5	0,920

Each row tests the null hypothesis that the Sample 1 and Sample 2 distributions are the same.
Group 1 = control + vehicle; Group 2 = TNBS + vehicle; Group 3 = TNBS + 0.1 mg/kg nafamostat;
Group 4 = TNBS + 1 mg/kg nafamostat; Group 5 = TNBS + 10 mg/kg nafamostat; Group 6 = Control + 10 mg/kg nafamostat

Supplementary file 1 - Details on statistical analysis of all data included in the manuscript

- p-values for microscopy

Sample 1 – Sample 2	p-value
6-1	0,857
6-2	0,014
6-5	0,003
6-4	0,001
6-3	0,000
1-2	0,023
1-5	0,006
1-4	0,002
1-3	0,001
2-5	0,637
2-4	0,412
2-3	0,266
5-4	0,728
5-3	0,523
4-3	0,770

Each row tests the null hypothesis that the Sample 1 and Sample 2 distributions are the same.
Group 1 = control + vehicle; Group 2 = TNBS + vehicle; Group 3 = TNBS + 0.1 mg/kg nafamostat;
Group 4 = TNBS + 1 mg/kg nafamostat; Group 5 = TNBS + 10 mg/kg nafamostat; Group 6 = Control + 10 mg/kg nafamostat

- p-values for MPO activity

Sample 1 – Sample 2	p-value
1-6	0,826
1-4	0,008
1-5	0,003
1-2	0,002
1-3	0,001
6-4	0,015
6-5	0,006
6-2	0,003
6-3	0,001
4-5	0,773
4-2	0,622
4-3	0,435
5-2	0,837
5-3	0,622
2-3	0,773

Each row tests the null hypothesis that the Sample 1 and Sample 2 distributions are the same.
Group 1 = control + vehicle; Group 2 = TNBS + vehicle; Group 3 = TNBS + 0.1 mg/kg nafamostat;
Group 4 = TNBS + 1 mg/kg nafamostat; Group 5 = TNBS + 10 mg/kg nafamostat; Group 6 = Control + 10 mg/kg nafamostat

3. mRNA expression control vs acute colitis

- Data are not normally distributed for tryptase and KLK8. Data are normally distributed for matriptase and uPA (Shapiro-Wilk test).
- Equality of variances cannot be assumed for tryptase. Equality of variances can be assumed for matriptase, uPA and KLK8 (Levene's test).
- Non-parametric testing with a Mann-Whitney U test was performed for tryptase and KLK8.
- Independent samples T-test was performed for matriptase and uPA.
- p-values

Variable	p-value
Tryptase	0,834
KLK8	0,248
Matriptase	0,001
uPA	1,000

4. VMR UAMC-00050 acute colitis

- Generalized Estimating Equations with Least Significant Difference post-hoc test was performed. Since there was a significant interaction between the factors "colitis_vs_control" and "dose", the pairwise comparisons between these factors were explored.
- Test of Model Effects

Variable	p-value
Colitis_vs_control	0,037
Dose	0,060
Pressure_AUC	0,000
Colitis_vs_control * Dose	0,008
Colitis_vs_control * Pressure_AUC	0,102
Dose * Pressure_AUC	0,083
Colitis_vs_control * Dose * Pressure_AUC	0,157

- Pairwise comparisons: Colitis_vs_control * Dose (with Bonferroni-Holm correction for multiple use of the vehicle-treated groups)

Variable 1	Variable 2	p-value
Control + vehicle	Control + 1 mg/kg UAMC-00050	1.000
Control + vehicle	Colitis + vehicle	0.040
Colitis + vehicle	Colitis + 0.1 mg/kg UAMC-00050	1.000
Colitis + vehicle	Colitis + 1 mg/kg UAMC-00050	0.001

5. VMR nafamostat acute colitis

- Generalized Estimating Equations with Least Significant Difference post-hoc test was performed. Since there was a significant interaction between the factors “colitis_vs_control” and “dose” as well as “colitis_vs_control” and “pressure_AUC”, the pairwise comparisons between these factors were explored.
- Test of Model Effects

Variable	p-value
Colitis_vs_control	0,133
Dose	0,129
Pressure_AUC	0,000
Colitis_vs_control * Dose	0,016
Colitis_vs_control * Pressure_AUC	0,696
Dose * Pressure_AUC	0,000
Colitis_vs_control * Dose * Pressure_AUC	0,086

- Pairwise comparisons: Colitis_vs_control * Dose (with Bonferroni-Holm correction for multiple use of the vehicle-treated groups)

Variable 1	Variable 2	p-value
Control + vehicle	Control + 10 mg/kg nafamostat	1,000
Control + vehicle	Colitis + vehicle	0,071
Colitis + vehicle	Colitis + 0.1 mg/kg nafamostat	1,000
Colitis + vehicle	Colitis + 1 mg/kg nafamostat	1,000
Colitis + vehicle	Colitis + 10 mg/kg nafamostat	0,065

6. VMR UAMC-00050 and nafamostat acetic acid model

- Generalized Estimating Equations with Least Significant Difference post-hoc test was performed. Since there was a significant interaction between the factors “colitis_vs_control” and “dose”, the pairwise comparisons between these factors were explored.
- Test of Model Effects

**Variable**	**p-value**
Colitis_vs_control	0,109
Dose	0,047
Pressure_AUC	0,000
Colitis_vs_control * Dose	0,001
Colitis_vs_control * Pressure_AUC	0,499
Dose * Pressure_AUC	0,330
Colitis_vs_control * Dose * Pressure_AUC	0,062

- Pairwise comparisons : Colitis_vs_control * Dose

**Variable 1**	**Variable 2**	**p-value**
Control + vehicle	Control + UAMC-00050	0,465
Control + vehicle	Control + nafamostat	0,145
Control + vehicle	Colitis + vehicle	<0,001
Colitis + vehicle	Colitis + UAMC-00050	<0,001
Colitis + vehicle	Colitis + nafamostat	0,009

7. Immunohistochemistry mast cell tryptase

- Data are not normally distributed (Shapiro-Wilk).
- Equality of variances cannot be assumed (Levene’s test)
- Non-parametric testing was performed on these data with Kruskal-Wallis test and pairwise comparisons
- p-values

**Sample 1 – Sample 2**	**p-value**
Control muscularis-Colitis muscularis	0,312
Control muscularis-Control submucosa	0,060
Control muscularis-Colitis submucosa	0,010
Control muscularis-Control mucosa	0,000
Control muscularis-Colitis mucosa	0,000
Colitis muscularis-Control submucosa	0,385
Colitis muscularis-Colitis submucosa	0,115
Colitis muscularis-Control mucosa	0,000
Colitis muscularis-Colitis mucosa	0,000
Control submucosa-Colitis submucosa	0,479
Control submucosa-Control mucosa	0,007
Control submucosa-Colitis mucosa	0,001
Colitis submucosa-Control mucosa	0,049
Colitis submucosa-Colitis mucosa	0,013
Control mucosa-Colitis mucosa	0,616

Each row tests the null hypothesis that the Sample 1 and Sample 2 distributions are the same.

8. Proteolytic activities colon

- Data are not normally distributed for TL_Boc, TL_Toc, CatG, NEI, PEI, KLK. Data are normally distributed for CL (Shapiro-Wilk).
- Equality of variances can be assumed for TL_Boc, TL_Toc, CL, CatG, NEI, KLK. Equality of variances cannot be assumed for PEI (Levene's test).
- Non-parametric testing was performed on these data to compare acute colitis + vehicle with acute colitis + UAMC-00050 using Mann Whitney U test.
- p-values

**Variable**	**p-value**
TL_Boc	0,245
TL_Toc	0,439
CL	0,197
CatG	0,697
NEI	0,053
PEI	0,028
KLK	0,090

9. Fecal protease activity azocasein assay

- Data are not normally distributed (Shapiro-Wilk).
- Equality of variances cannot be assumed (Levene's test).
- Non-parametric testing was performed on these data to compare control with acute colitis group with Wilcoxon matched-pairs signed-rank test
- p-value: 0,006

10. Proteolytic activities fecal samples

- Data are not normally distributed for CL, NEI, CatG. Data are normally distributed for TL_Boc, TL_Toc, PEI, KLK (Shapiro-Wilk).
- Equality of variances can be assumed for TL_Boc, TL_Toc, PEI, NEI, KLK. Equality of variances cannot be assumed for CL and CatG (Levene's test).
- Two-way ANOVA was performed for TL_Boc, TL_Toc, PEI, KLK. No interaction effect was found. A significant effect of condition was found for PEI and KLK. A significant effect for treatment was found for PEI. Unpaired T-tests + Bonferroni correction
- Nonparametric testing was performed for CL, NEI, CatG. Tested for main effects via reduced model split file and Mann Whitney U test + Bonferroni correction
- p-values Two-Way ANOVA - condition * treatment

**Variable**	**p-value**
TL_Boc	0,692
TL_Toc	0,622
PEI	0,929
KLK	0,809

Supplementary file 1 - Details on statistical analysis of all data included in the manuscript

- p-values Two-Way ANOVA - condition

Variable	p-value
TL_Boc	0,898
TL_Toc	0,937
PEI	0,021
KLK	0,019

- p-values Two-Way ANOVA – treatment

Variable	p-value
TL_Boc	0,133
TL_Toc	0,066
PEI	0,000
KLK	0,127

- p-values unpaired T-test – effect of treatment in control animals

Variable	p-value
PEI	0,004

- p-values unpaired T-test – effect of treatment in acute colitis animals

Variable	p-value
PEI	0,006

- p-values unpaired T-test – effect of condition in vehicle-treated animals

Variable	p-value
PEI	0,014
KLK	0,118

- p-values unpaired T-test – effect of condition in UAMC-treated animals

Variable	p-value
PEI	0,184
KLK	0,075

- p-values Mann Whitney U – effect of treatment in control animals

Variable	p-value
CL	0,110
CatG	0,600
NEI	1,000

- p-values Mann Whitney U – effect of treatment in acute colitis animals

Variable	p-value
CL	0,218
CatG	0,200
NEI	0,056

- p-values Mann Whitney U – effect of condition in vehicle-treated animals

**Variable**	**p-value**
CL	0,156
CatG	0,032
NEI	0,332

- p-values Mann Whitney U – effect of condition in UAMC-treated animals

**Variable**	**p-value**
CL	0,218
CatG	0,400
NEI	1,000
